# Supplementary material for: Functional orderly topography of brain networks associated with gene expression heterogeneity
Source: Commun Biol. 2022 Oct 11;5:1083. doi: 10.1038/s42003-022-04039-8 (PMC9554040; doi:10.1038/s42003-022-04039-8)

Supplementary Methods

Constructing gene expression networks in samples of human brains. The gene expression data were downloaded from the Allen Institute for Brain Science (http://human.brain-map.org/), include data from six adult brains (two contributed both hemispheres, and four contributed one hemisphere), for a total of 3,702 brain samples[1]. If two genes were expressed in a given brain sample and were available in the integrated human protein interaction network, then both genes were included in the gene expression network of the sample. By integrating the expression data and large-scale protein interaction data, we established gene expression networks for 2,703 samples from six human brains. Because the sample size of Brain #1 was the greatest of the six brains, we considered Brain #1 a typical case to illustrate the main results and used the results of the other brains as a reference in the following analysis.

The integrated human protein interaction dataset. We established a human protein interaction dataset by combining protein interaction data from multiple databases. First, we merged the global human physical protein interaction network from previous material[2] with the iRefIndex database[3], which extracted original protein interactions from the BIND, BioGRID, CORUM, DIP, HPRD, IntAct, MINT, MPact, MPPI and OPHID databases. To ensure the reliability of the protein interactions, only interactions supported by at least one piece of direct experimental evidence were included to demonstrate the physical association between two human proteins. By unifying protein accessions and deleting redundant interactions, we finally established an integrated protein interaction dataset containing 13,509 proteins and 172,848 interactions (Supplementary Table 3).

Computation of the evolutionary rate. The evolutionary rate is a measurement used to quantify the speed of evolutionary change. We calculated the dN/dS values for all genes expressed in the

human brains to characterize their evolutionary rates. The synonymous and non-synonymous substitution rates between human and mouse were obtained from Ensembl (http://www.ensembl.org/biomart/martview/).

Small-world structure of gene expression network. Previous studies on the macro-level brain connectome have demonstrated that the human brain has a small-world structure with a characteristic path length that is lower than that of the regular network and a clustering coefficient that is greater than that of a random network[4, 5]. At the micro level, we expected that the gene expression networks of all brain samples would exhibit the small-world property. We generated populations of regular networks and random graphs that preserved the same number of nodes and edges in the gene expression networks and extracted their mean path length and average clustering coefficient. We found that the gene expression networks have shorter path lengths and larger clustering coefficients compared to those of the random and regular networks (Supplementary Fig. 2), confirming the small-world property of the gene expression networks in human brains.

Topological properties of the networks. The most elementary characteristic of a node is its degree (or connectivity), k, which provides information regarding the number of links that a node has to other nodes. The mean path length represents the average of the shortest paths between all pairs of nodes and offers a measure of a network's overall navigability[6]. An undirected network with n nodes and l links is characterized by an average degree, $k=l/n$. In addition, the average clustering coefficient characterizes the overall tendency of the nodes to form clusters or groups[7]. The closer the local clustering coefficient is to 1, the more likely that the network will form clusters. The betweenness centrality is a measure of the importance of a node in a network, i.e., the relative importance of a node to the network. The betweenness centrality is equal to the

fraction of the shortest paths that pass through a node and is counted over the shortest paths among all pairs of nodes.

The eigen entropy of networks was defined as the entropy of the normalized largest eigen vector of an adjacency matrix[8]. For a network with *n* genes, its adjacency matrix can be represented as $A_{ij}\left(i, j = 1 \sim n\right)$, which describes the interacting relationships of the genes in the networks. If gene *i* interacts with gene *j*, $A_{ij} = 1$; otherwise, $A_{ij} = 0$. We assumed that the eigen vector of matrix A corresponding to the largest eigen value λ_k is r_k. By dividing by the sum of all eigen vectors, we normalized this vector to obtain its energy concentration, i.e., $I_k = \frac{r_k}{\sum_{i=1}^{n} r_k}$.

The eigen entropy of this network is defined as $S_p = -\sum_{k=1}^{n} I_k \ln I_k$, which can reflect the degree of ordering in each network. A smaller eigen entropy of a network corresponds to a higher degree of ordering of the network.

Integration of brain-wide gene expression dataset from Allen Human Brain Atlas. We combined the gene expresion dataset across all six brains using the typical workflow for processing brain-wide transcriptomic data described in previous paper[19]. By mapping the samples to cerebral cortex of human brains, we obtain an integrated dataset including 15,745 genes expressed in 1280 samples. The following probe filtering criteria were applied during the process: 1) the probe-to-gene annotations were updated using Re-Annotator package; 2) probes where expression measures do not exceed the background in more than 50% samples were removed; 3) a representative probe for a gene was selected based on the highest intensity; 4) applying limma batch effect removal on cross-subject aggregated data, followed by the scaled robust sigmoid normalization. After normalization, samples have no inter-individual differences in gene

expression.

Functional connectivity of human brains. Functional connectivity was analyzed using resting-state fMRI data from fifty randomly selected subjects from the Human Connectome Project (HCP, http://www.humanconnectome.org/documentation/S500/). The HCP minimal preprocessing pipeline was used for the resting-state fMRI data[9]. This pipeline includes artifact removal, motion correction, and registration to a standard space. The fMRI data were then processed using previously described procedures[10] as follows: 1) spatial smoothing using a 6-mm full-width half-maximum Gaussian kernel; 2) normalization of the global mean signal intensity across runs; 3) linear detrending and bandpass temporal filtering (0.01~0.08 Hz); and 4) regression of nuisance variables, including the six parameters obtained by rigid body head motion correction, ventricular and white matter signals, and the first temporal derivatives of all the above items. The AAL atlas, which divided the whole brain into 116 regions, was used for the region-to-region functional connectivity measures in the current study[11]. All AAL regional masks were generated using the free software WFU_PickAtlas (Version 2.0, http://www.ansir.wfubmc.edu). We evaluated the functional connectivity between each pair of regional averaged time courses using Pearson's correlation coefficient. Significant functional correlations were selected using one-sample *t*-tests ($P<0.05$, Bonferroni correction), resulting in the binary 116×116 symmetric connectivity matrix C of the functional connectivity network in human brains (Supplementary Table 7).

To match the functional connectivity network in the human brains, we mapped 2,703 samples containing transcriptional data to 116 brain regions. By averaging the attribute values of samples of the same region, we obtained the mean gene expression characteristics and network properties

of the brain regions (104, 104, 50, 59, 60, and 62 regions mapped to Brains #1-6, respectively).

The gene expression network in the mouse brain. We established the expression networks in mouse brain structures based on the Allen Institute mouse brain atlas, which offers finely sampled whole-genome expression data[12]. According to the Allen Reference Atlas, a 56-day-old male C57BL/6J mouse brain was partitioned into 73 structures and 12 regions. We computed the expression levels of 719,905 genes in 73 brain structures contained in the coronal planes. Based on the expression intensity in each voxel, we obtained the expression levels of genes in 73 structures by averaging across all voxels in the brain structures. The expression levels in the mouse in situ hybridization data from the Allen Mouse Brain Atlas were quantified using a metric called expression energy (fraction of stained volume × the average intensity of staining) as previously described[12]. In total, 2,873 genes were found to be expressed in at least one structure by selecting genes with fractions expressing pixels above 0.02 to omit genes with extremely low expression. We downloaded 33,145 protein interactions among 8,499 mouse gene products from the BIOGRID database[13]. By integrating the gene expression data and protein interactions in the mouse brain, we established interaction networks in 73 structures of the adult mouse brain.

We identified 570 HKGs that were expressed in 72 or 73 structures and 360 specific genes that were expressed in one or two structures. Regarding the genes expressed in more structures in the mouse brain, their homologous genes in humans tended to be more widely expressed in the human brains (R=0.23, P<0.01). In particular, most of the HKGs (33.68-79.65%) in the mouse brain are homologous to the HKGs in the human Brains #1-6. The percentage of HKGs in gene expression networks of the mouse brain was positively correlated with the average degree (R=0.63, P<0.01) and clustering coefficient (R=0.18, P<0.01) and negatively correlated with the mean path length

(*R*=-0.23, *P*<0.01) and eigen entropy (*R*=-0.89, *P*<0.01).

Functional connectivity of mouse brains. Resting-state fMRI data of anesthetized mice were collected from fifty male C57BL/6J mice (Janvier, Le Genest-St Isle, France) between 10 and 13 weeks old weighing 30.6±1.9 g (mean + SD), which are publicly available on the central.xnat.org repository in Analyze 7.5 format (Project ID: fMRI_ane_mouse). Two mice were excluded due to normalization failure. Preprocessing was performed using SPM8 (Wellcome Trust Centre for Neuroimaging, London, UK, http://www.fil.ion.ucl.ac.uk/spm). For each mouse, the first 10 time points were dismissed to account for magnetic saturation. Then, the following steps were performed: 1) slice timing correction; 2) motion correction; 3) normalization with an in-house EPI template (0.2-mm isotropic voxels); 4) spatial smoothing using a 0.4-mm full width half-maximum Gaussian kernel; 5) linear detrending and band-pass temporal filtering (0.01–0.3 Hz); 6) regression of nuisance variables, including the six parameters obtained by rigid body head motion correction, global signals, and their first temporal derivatives. The functional connectivity between each pair of regional averaged time courses was evaluated using Pearson's correlation coefficient. Significant functional correlations were selected to obtain the binary 22×22 symmetric connectivity matrix of the functional connectivity network in mouse brains (Supplementary Table 8). We mapped the functional connectivity data of the mouse brains to 22 brain regions and computed the degree of each region in the functional connectivity network.

Gene expression network of adult rhesus macaque brains. We downloaded the original gene expression CEL files of the three 48 month specimens generated serially across a complete hemisphere from adult rhesus macaque from the NIH Blueprint Non-Human Primate (NHP) Atlas (http://www.blueprintnhpatlas.org) and extracted normalized and processed expression levels of

all genes based on R language program. Neocortex include rostral cingulate cortex, medial orbitofrontal cortex, caudal orbitofrontal cortex, dorsolateral prefrontal cortex and V1,V2, archicortex include Hipp, and subcortex include AMY, Tu, striatum and globus pallidus. Interaction data of rhesus macaque is obtained from STRING database (version 10.5). Based on the expression data and protein interactions in the rhesus macaque brain, we established gene expression networks and analyzed their gene expression characteristics and topological properties.

Functional connectivity of adult rhesus macaque brains. The monkey fMRI data were from PRIME-DE, an open resource for non-human primate imaging (http://fcon_1000.projects.nitrc.org/indi/indiPRIME.html)[14]. The image data were collected from a group of 12 male anesthetized rhesus macaque monkeys (Macaca mulatta), aged between 4 and 8 years, and weighed 6.1-11.8 kg in University of Western Ontario[15,16].

The resting-state experiments were conducted on a 7T MRI scanner equipped with a 40-cm gradient coil set of 80 mT/m strength (Siemens, Erlangen, Germany), and a custom-made 24-channel phased array receive coil with an 8-channel transmit coil was used. Resting-state images were acquired using a 2-dimensional multiBand and EPI sequence: repetition time/echo time = 1000/18 ms, flip angle = 40°, field of view = 96×96 mm, voxel size = 1.0 * 1.0 * 1.0 mm, and number of slices = 42. Totally, 600 time frames were collected for each run, and each monkey underwent three or four runs.

Preprocessing was performed using SPM8 (Wellcome Trust Centre for Neuroimaging, London, UK, http://www.fil.ion.ucl.ac.uk/spm). For each monkey, the first 10 time points were dismissed to account for magnetic saturation. Then, the following steps were then performed: 1) slice timing

correction; 2) motion correction; 3) normalization with the INIA19 template (1.0-mm isotropic voxels)[17]; 4) spatial smoothing using a 2-mm full width half-maximum Gaussian kernel; 5) linear detrend and band-pass temporal filtering (0.01–0.3 Hz); 6) regression of nuisance variables, including the six parameters obtained by rigid body head motion correction, global signal, and their first temporal derivatives.

Based on D99 template of macaque brain[18], the functional connectivity between each pair of regional averaged time courses was evaluated using Pearson's correlation coefficient. Significant functional correlations (r>0.15) were selected to obtain the binary 304×304 symmetric connectivity matrix of the functional connectivity network in macaque brains (Supplementary Table 9). We mapped the functional connectivity data of the macaque brains to 304 brain structures and computed the degree of each structure in the functional connectivity network.

The description of structures in rhesus macaque

rCG: rostral cingulate cortex; mOF: medial orbitofrontal cortex; cOF: caudal orbitofrontal cortex; dlPF: dorsolateral prefrontal cortex; DFC/VFC: dorsolateral/ventrolateral prefrontal cortex; Pir: parietal cortex; PAC: periamygdaloid cortex; TE: inferotemporal cortex area; F: aguranular frontal cortex; Cg: cingulate cortex; G: gustatory cortex; EC: enrothinal cortex; RSC: retrosplenial cortex; A: auditory cortex; S: somatosensory areas; INS: insula; V1: visual area 1; V2: visual area 2; V3: visual area 3; V4: visual area 4; OT: olfactory tubercle; Hipp: hippocampal region; GP: globus pallidus; AMY: amygdala.

The influence of methodological variability on the analyses results

Considering that choice of processing steps and parameters can have a potential influence on the statistical outcomes of research with the AHBA, we used abagen as a tool to investigate the

influence of methodological variability on our results. We varied the methods of gene normalization, sample normalization and probe selection and took other parameters as default values and computed the heterogeneity index of brain regions based on the Desikan-Killiany atlas. The results are consistent under different standardizing workflows. In 10 distinct processing pipelines, the heterogeneity of gene expression in the neocortex was found to be significantly greater than those in the archicortex and subcortex, with the regions in neocortex tend to have lower standard errors of gene expression levels than those in archicortex and subcortex.

Supplementary References

1. Hawrylycz, M. J. et al. An anatomically comprehensive atlas of the adult human brain transcriptome. *Nature* **489(7416)**, 391-399 (2012).
2. Bossi, A. & Lehner, B. Region specificity and the human protein interaction network. *Molecular Systems Biology* **5**, 260 (2009).
3. Razick, S., Magklaras, G. & Donaldson, I. M. iRefIndex: a consolidated protein interaction database with provenance. *BMC Bioinformatics* **9**, 405 (2008).
4. Achard, S., Salvador, R., Whitcher, B., Suckling, J. & Bullmore, E. A resilient, low-frequency, small-world human brain functional network with highly connected association cortical hubs. *J. Neurosci.* **26,** 63-72 (2006).
5. Liao, X., Vasilakos, A. V. & Yong, H. Small-world human brain networks: Perspectives and challenges. *Neurosci. Biobehav. Rev.* **77**, 286-300 (2017).
6. Barabasi, A. L. & Oltvai, Z. N. Network biology: understanding the cell's functional organization. *Nature Reviews* **5(2)**, 101-113 (2004).
7. Milo, R., Shen-Orr, S. S., Itzkovitz, S., Kashtan, N. & Alon, U. Network motifs: simple building blocks of complex networks. *Science* **298**, 824-827 (2002).
8. Fan, Y. et al. Lifespan development of the human brain revealed by large-scale network eigen-entropy. *Entropy* **19**, 471 (2017).
9. Craddock, R. C., James, G. A., Holtzheimer, P. E., Hu, X. P. & Mayberg, H. S. A whole brain fMRI atlas generated via spatially constrained spectral clustering. *Hum. Brain Mapp*. **33**, 1914-1928 (2012).
10. Zeng, L. L. et al. Neurobiological basis of head motion in brain imaging. *PNAS.* **111(16)**, 6058-6062 (2014).
11. Tzourio-Mazoyer, N. et al. Automated anatomical labeling of activations in SPM using a macroscopic anatomical parcellation of the MNI MRI single-subject brain. *Neuroimage* **15**, 273-289 (2012).
12. Ng, L. et al. An anatomic gene expression atlas of the adult mouse brain. *Nature Neuroscience* **12(3)**, 356-362 (2009).
13. Stark, C. et al. BioGRID: A General Repository for Interaction Datasets. *Nucleic Acids Res.* **34**, D535-D539 (2006).
14. Michael, P. M. et al. An open resource for non-human primate imaging. *Neuron* **100**, 61-74 (2018).
15. Gilbert, K. M., Gati, J. S., Barker, K., Everling, S. & Menon, R. S. Optimized parallel transmit and receive

radiofrequency coil for ultrahigh-field MRI of monkeys. *Neuroimage* **125**, 153-161 (2016).

16. Ghahremani, M., Hutchison, R. M., Menon, R. S. & Everling, S. Frontoparietal functional connectivity in the common marmoset. *Cerebral Cortex* **27(8)**, 3890-3905 (2017).
17. Torsten, R. et al. The INIA19 template and NeuroMaps atlas for primate brain image parcellation and spatial normalization. *Frontiers in Neuroinformatics* **6**, 7 (2012).
18. Reveley, C. et al. Three-dimensional digital template atlas of the macaque brain. *Cereb Cortex* **27(9)**, 4463-4477 (2017).
19. Arnatkevic, I. A., Fulcher, B. D. & Fornito, A. A practical guide to linking brain-wide gene expression and neuroimaging data. *Neuroimage* **189**, 353-367 (2019).

Supplementary Figures

Supplementary Figure 1 The percentage of HKGs mapped to the brain samples of Brain #1-6.

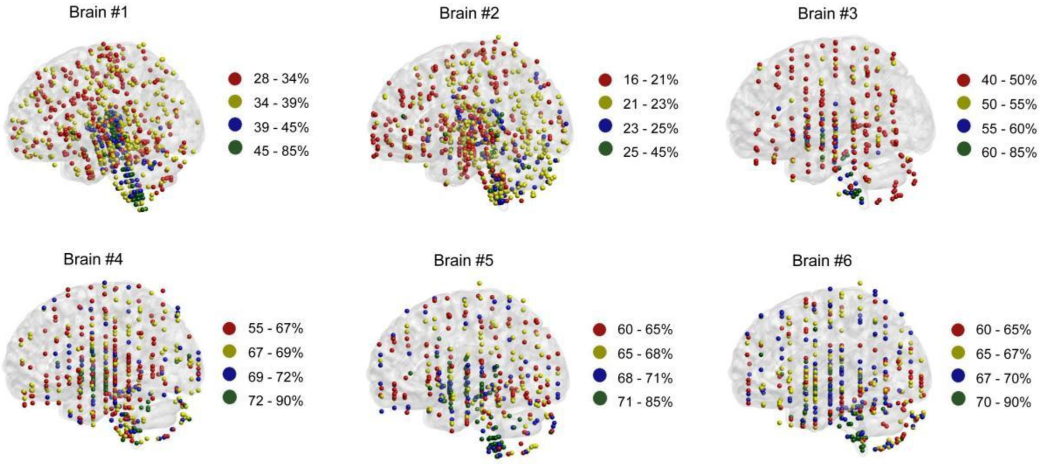

Supplementary Figure 2 Correlation of network properties of gene expression and functional connectivity.

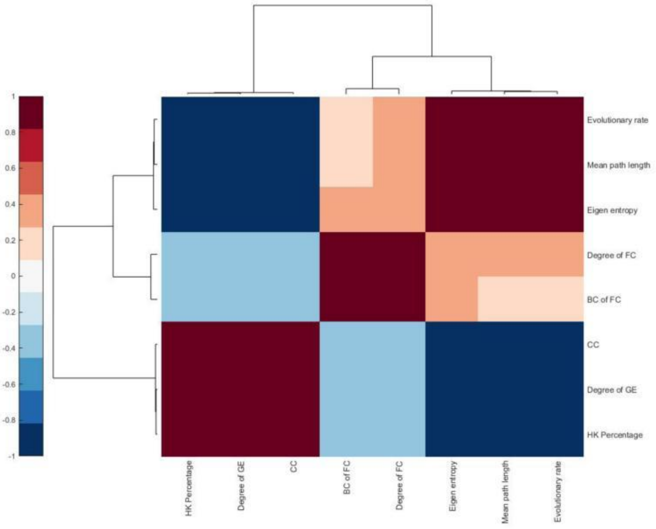

Supplementary Figure 3 The percentage of HKGs mapped to the isocortex of mouse brain.

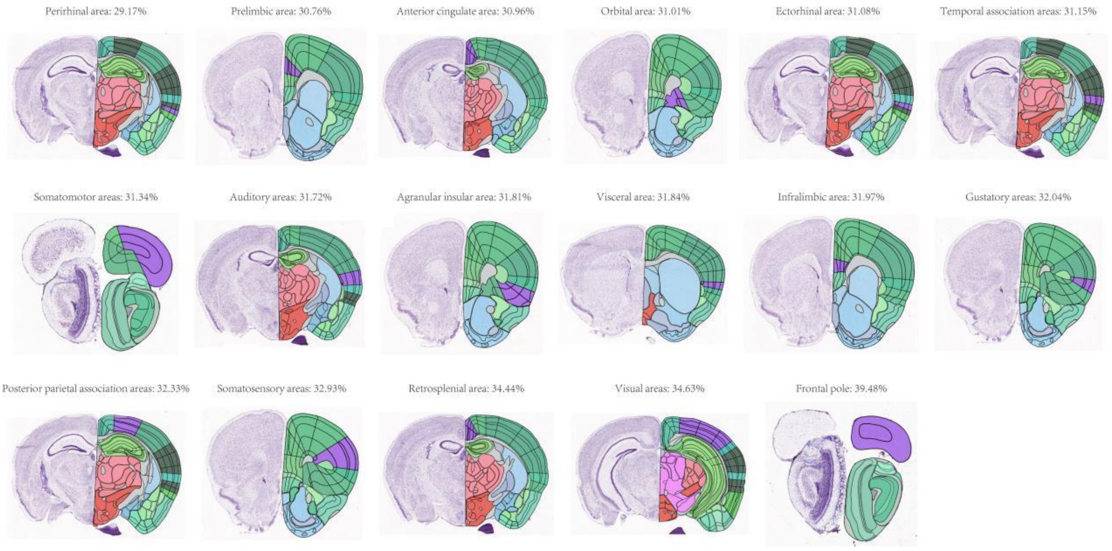

Supplementary Figure 4 The mean value and standard deviation of topological indices in the neocortex, archicortex and subcortex of Brain #1-6.

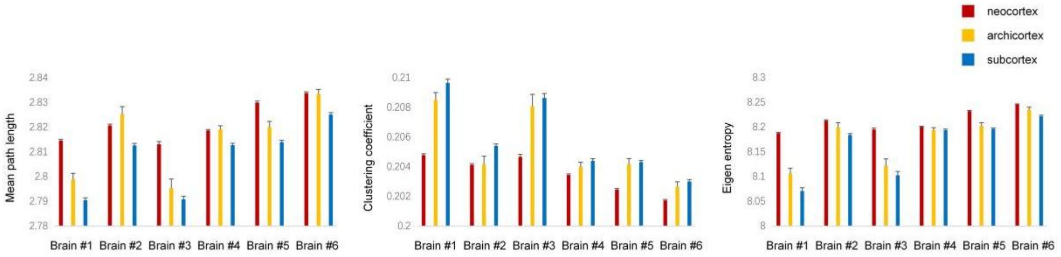

Supplement: Supplementary file 2 — Supplementary Information [file 42003_2022_4039_MOESM2_ESM.pdf]
