# Supplementary material for: Functional orderly topography of brain networks associated with gene expression heterogeneity
Source: Commun Biol. 2022 Oct 11;5:1083. doi: 10.1038/s42003-022-04039-8 (PMC9554040; doi:10.1038/s42003-022-04039-8)
Supplement: Supplementary file 3 — Description of Additional Supplementary Files [file 42003_2022_4039_MOESM3_ESM.pdf]

Description of Additional Supplementary Files

File name: Supplementary Data 1
Description: The gene expression, HKG and specific gene percentage of samples in six human brains

File name: Supplementary Data 2
Description: The network properties of samples in six human brains

File name: Supplementary Data 3
Description: The human protein interaction dataset

File name: Supplementary Data 4
Description: The heterogeneity indices of structures in the mouse brain

File name: Supplementary Data 5
Description: The heterogeneity indices of human brain regions in different processing pipelines

File name: Supplementary Data 6
Description: The heterogeneity indices of structures in the rhesus macaque brain

File name: Supplementary Data 7
Description: The Z-scores and P-values of the functional connectivity network in human brains

File name: Supplementary Data 8
Description: The Z-scores and P-values of the functional connectivity network in the mouse brain

File name: Supplementary Data 9
Description: The Z-scores of the functional connectivity network in the rhesus macaque brain
