# Supplementary material for: Functional orderly topography of brain networks associated with gene expression heterogeneity
Source: Commun Biol. 2022 Oct 11;5:1083. doi: 10.1038/s42003-022-04039-8 (PMC9554040; doi:10.1038/s42003-022-04039-8)
Supplement: Supplementary file 13 — Reporting Summary [file 42003_2022_4039_MOESM13_ESM.pdf]

nature portfolio

Corresponding author(s): liuwei

Last updated by author(s): Sep 9, 2022

Reporting Summary

Nature Portfolio wishes to improve the reproducibility of the work that we publish. This form provides structure for consistency and transparency in reporting. For further information on Nature Portfolio policies, see our Editorial Policies and the Editorial Policy Checklist.

Statistics

For all statistical analyses, confirm that the following items are present in the figure legend, table legend, main text, or Methods section.

n/a	Confirmed	
☐	☒	The exact sample size (*n*) for each experimental group/condition, given as a discrete number and unit of measurement
☐	☒	A statement on whether measurements were taken from distinct samples or whether the same sample was measured repeatedly
☐	☒	The statistical test(s) used AND whether they are one- or two-sided<br>*Only common tests should be described solely by name; describe more complex techniques in the Methods section.*
☐	☒	A description of all covariates tested
☐	☒	A description of any assumptions or corrections, such as tests of normality and adjustment for multiple comparisons
☐	☒	A full description of the statistical parameters including central tendency (e.g. means) or other basic estimates (e.g. regression coefficient) AND variation (e.g. standard deviation) or associated estimates of uncertainty (e.g. confidence intervals)
☐	☒	For null hypothesis testing, the test statistic (e.g. *F*, *t*, *r*) with confidence intervals, effect sizes, degrees of freedom and *P* value noted<br>*Give P values as exact values whenever suitable.*
☐	☒	For Bayesian analysis, information on the choice of priors and Markov chain Monte Carlo settings
☐	☒	For hierarchical and complex designs, identification of the appropriate level for tests and full reporting of outcomes
☐	☒	Estimates of effect sizes (e.g. Cohen's *d*, Pearson's *r*), indicating how they were calculated

Our web collection on statistics for biologists contains articles on many of the points above.

Software and code

Policy information about availability of computer code

Data collection	No software was used.
Data analysis	The Matlab code used to analyze the gene expression characteristics and the topological properties of networks detailed in this study were provided on the github.

For manuscripts utilizing custom algorithms or software that are central to the research but not yet described in published literature, software must be made available to editors and reviewers. We strongly encourage code deposition in a community repository (e.g. GitHub). See the Nature Portfolio guidelines for submitting code & software for further information.

Data

Policy information about availability of data

All manuscripts must include a data availability statement. This statement should provide the following information, where applicable:

- Accession codes, unique identifiers, or web links for publicly available datasets
- A description of any restrictions on data availability
- For clinical datasets or third party data, please ensure that the statement adheres to our policy

The expression data used in this study are available via the Allen Institute for Brain Atlas (see http://brain-map.org/). The interactions in human, rhesus macaque and mouse are available in Supplementary Table 7, STRING and BIOGRID database. Resting-state fMRI data of human, rhesus macaque, and mouse can be found in http://www.humanconnectome.org/documentation/S500/, http://fcon_1000.projects.nitrc.org/indi/indiPRIME.html, and central.xnat.org, respectively. The authors declare that the data supporting the findings of this study are available within the article, its supplementary information, and upon request.

Field-specific reporting

Please select the one below that is the best fit for your research. If you are not sure, read the appropriate sections before making your selection.

☒ Life sciences ☐ Behavioural & social sciences ☐ Ecological, evolutionary & environmental sciences

For a reference copy of the document with all sections, see nature.com/documents/nr-reporting-summary-flat.pdf

Life sciences study design

All studies must disclose on these points even when the disclosure is negative.

Sample size	The gene expression data were downloaded from the Allen Institute for Brain Science, include data from six adult brains (two contributed both hemispheres, and four contributed one hemisphere), for a total of 3,702 brain samples.
Data exclusions	No data was excluded from the analysis.
Replication	The results obtained from the different resolution networks and different individuals are consistent, indicating that such gene expression pattern in brains is robust and inherent.
Randomization	Because the sample size of Brain #1 was the greatest of the six brains, we considered Brain #1 a typical case to illustrate the main results and used the results of the other brains as a reference in the following analysis.
Blinding	Blinding is not possible, because the expression data was collected from postmortem human brain tissue.

Reporting for specific materials, systems and methods

We require information from authors about some types of materials, experimental systems and methods used in many studies. Here, indicate whether each material, system or method listed is relevant to your study. If you are not sure if a list item applies to your research, read the appropriate section before selecting a response.

Materials & experimental systems

n/a	Involved in the study	
☒	☐	Antibodies
☒	☐	Eukaryotic cell lines
☒	☐	Palaeontology and archaeology
☒	☐	Animals and other organisms
☒	☐	Human research participants
☒	☐	Clinical data
☒	☐	Dual use research of concern

Methods

n/a	Involved in the study	
☒	☐	ChIP-seq
☒	☐	Flow cytometry
☒	☐	MRI-based neuroimaging
